# Genome Sequence of a Strain of the Human Pathogenic Bacterium *Pseudomonas alcaligenes* That Caused Bloodstream Infection

**DOI:** 10.1128/genomeA.00919-13

**Published:** 2013-10-31

**Authors:** Masato Suzuki, Satowa Suzuki, Mari Matsui, Yoichi Hiraki, Fumio Kawano, Keigo Shibayama

**Affiliations:** Department of Bacteriology II, National Institute of Infectious Diseases, Musashimurayama, Tokyo, Japana; Department of Pharmacy, National Hospital Organization Kumamoto Medical Center, Chuo-ku, Kumamoto, Japanb; Department of Hematology, National Hospital Organization Kumamoto Medical Center, Chuo-ku, Kumamoto, Japanc

## Abstract

*Pseudomonas alcaligenes*, a Gram-negative aerobic bacterium, is a rare opportunistic human pathogen. Here, we report the whole-genome sequence of *P. alcaligenes* strain MRY13-0052, which was isolated from a bloodstream infection in a medical institution in Japan and is resistant to antimicrobial agents, including broad-spectrum cephalosporins and monobactams.

## GENOME ANNOUNCEMENT

*Pseudomonas alcaligenes* is a Gram-negative aerobic rod belonging to the bacterial family *Pseudomonadaceae* and is a common inhabitant of soil and water. A recent study showed that *P. alcaligenes* is useful as a microbial inoculant for the biodegradation of toxic polycyclic aromatic hydrocarbons (1). *P. alcaligenes* has also been known as a rare opportunistic human pathogen (2). Based on 16S rRNA gene sequence analysis, *P. alcaligenes* was classified in the *Pseudomonas aeruginosa* group (3). However, little is known about the clinical importance of *P. alcaligenes* and its virulence factors, mainly because of the difficulties in identifying and distinguishing this bacterium from closely related *Pseudomonas* species in medical settings.

In this report, we announce the availability of the first draft genome sequence of *P. alcaligenes*. *P. alcaligenes* strain MRY13-0052 was recovered from a bloodstream infection in a medical institution in Japan in 2013 and was resistant to broad-spectrum cephalosporins and monobactams. Whole-genome shotgun (WGS) sequencing of strain MRY13-0052 was performed using the Roche 454 pyrosequencing platform (500-bp insert size). The reads were assembled *de novo* using Newbler Assembler version 2.3 (Roche). The draft genome sequence of MRY13-0052 consists of 237 contigs, yielding a total of 6,876,944 bp with an N_50_ contig size of 64,175 bp. The mean G+C content was 65.8%. A total of 6,190 coding DNA sequences were identified by the RAST server (http://rast.nmpdr.org) (4). The MRY13-0052 strain carried three class C β-lactamase genes that might confer resistance to β-lactam antibiotics. Any other acquired antimicrobial resistance genes in the WGS data were not detected using a Web-based tool, ResFinder version 1.3 (http://cge.cbs.dtu.dk/services/ResFinder/) (5).

Bacterial pathogens frequently use protein secretions to interact with their hosts. MRY13-0052 contains the type VI secretion system (T6SS) gene cluster and three genes that encode VgrG (valine glycine repeat G) translocator proteins (6). The T6SS, which is conserved among *Pseudomonas* species (7), delivers effectors into neighboring organisms, including bacteria and mammalian cells, leading to cytotoxicity and cell death in the targets (6). The MRY13-0052 strain furthermore contains a set of genes that encode proteins homologous to *P. aeruginosa* Tse1 (type VI effector 1) and Tsi1 (type VI immunity 1) (8) (66.9% and 48.8% identities, respectively). On the other hand, MRY13-0052 is devoid of the virulence-associated type III secretion system (T3SS) gene cluster, whereas a T3SS is the major virulence factor in animal and plant pathogenic *Pseudomonas* species, *P. aeruginosa*, and *Pseudomonas syringae* (9, 10). These data suggest that the T6SS might be an important virulence determinant in *P. alcaligenes* and that *P. alcaligenes* might partially share the same T6SS-dependent effector and immunity system with *P. aeruginosa*.

A more detailed report of the virulence phenotype of *P. alcaligenes* MRY13-0052 will be included in a future publication. A genome-wide comparison of *P. alcaligenes* with related *Pseudomonas* species, such as *P. aeruginosa*, *Pseudomonas mendocina*, and *Pseudomonas pseudoalcaligenes*, will facilitate additional comprehensive bioinformatic and phylogenetic analyses, thus expanding our understanding of fatal nosocomial infections caused by these opportunistic human pathogens.

### Nucleotide sequence accession numbers.

The WGS projects for *P. alcaligenes* MRY13-0052 have been deposited at DDBJ/EMBL/GenBank under the accession no. BATO00000000. The version described in this paper is the first version, BATO01000000.

## References

[1] O’Mahony MM Dobson AD Barnes JD Singleton I 2006 The use of ozone in the remediation of polycyclic aromatic hydrocarbon contaminated soil. Chemosphere 63:307–314 16153687 10.1016/j.chemosphere.2005.07.018

[2] Martino P Micozzi A Venditti M Gentile G Girmenia C Raccah R Santilli S Alessandri N Mandelli F 1990 Catheter-related right-sided endocarditis in bone marrow transplant recipients. Rev. Infect. Dis. 12:250–257 2330480 10.1093/clinids/12.2.250

[3] Anzai Y Kim H Park JY Wakabayashi H Oyaizu H 2000 Phylogenetic affiliation of the pseudomonads based on 16S rRNA sequence. Int. J. Syst. Evol. Microbiol. 50 (Pt 4):1563–1589 10939664 10.1099/00207713-50-4-1563

[4] Aziz RK Bartels D Best AA DeJongh M Disz T Edwards RA Formsma K Gerdes S Glass EM Kubal M Meyer F Olsen GJ Olson R Osterman AL Overbeek RA McNeil LK Paarmann D Paczian T Parrello B Pusch GD Reich C Stevens R Vassieva O Vonstein V Wilke A Zagnitko O 2008 The RAST server: rapid annotations using subsystems technology. BMC Genomics 9:75. 10.1186/1471-2164-9-75 18261238 PMC2265698

[5] Zankari E Hasman H Cosentino S Vestergaard M Rasmussen S Lund O Aarestrup FM Larsen MV 2012 Identification of acquired antimicrobial resistance genes. J. Antimicrob. Chemother. 67:2640–2644 22782487 10.1093/jac/dks261 PMC3468078

[6] Silverman JM Brunet YR Cascales E Mougous JD 2012 Structure and regulation of the type VI secretion system. Annu. Rev. Microbiol. 66:453–472 22746332 10.1146/annurev-micro-121809-151619 PMC3595004

[7] Barret M Egan F Fargier E Morrissey JP O’Gara F 2011 Genomic analysis of the type VI secretion systems in *Pseudomonas* spp.: novel clusters and putative effectors uncovered. Microbiology 157:1726–1739 21474537 10.1099/mic.0.048645-0

[8] Russell AB Hood RD Bui NK LeRoux M Vollmer W Mougous JD 2011 Type VI secretion delivers bacteriolytic effectors to target cells. Nature 475:343–347 21776080 10.1038/nature10244 PMC3146020

[9] Hauser AR 2009 The type III secretion system of *Pseudomonas aeruginosa*: infection by injection. Nat. Rev. Microbiol. 7:654–665 19680249 10.1038/nrmicro2199 PMC2766515

[10] Lindeberg M Cunnac S Collmer A 2012 *Pseudomonas syringae* type III effector repertoires: last words in endless arguments. Trends Microbiol. 20:199–208 22341410 10.1016/j.tim.2012.01.003

